# A participant-randomized pilot hybrid II trial of group cognitive processing therapy for incarcerated persons with posttraumatic stress and substance use disorder symptoms: study protocol and rationale

**DOI:** 10.1186/s40352-022-00192-8

**Published:** 2022-10-01

**Authors:** Melissa J. Zielinski, Mollee K. Steely Smith, Debra Kaysen, James P. Selig, Nickolas D. Zaller, Geoffrey Curran, JoAnn E. Kirchner

**Affiliations:** 1 University of Arkansas for Medical Sciences, Little Rock, AR USA; 2 University of Arkansas, Fayetteville, USA; 3 Stanford University, Stanford, USA; 4 Central Arkansas Veterans Healthcare System, North Little Rock, USA

**Keywords:** Prison, Incarceration, Posttraumatic stress disorder, Substance use disorder, Implementation facilitation, Cognitive processing therapy

## Abstract

**Background:**

Trauma exposure and drug addiction go hand-in-hand for the 2.17 million people who are incarcerated in US prisons; prevalence of both exceed 80% among this population. This manuscript describes the rationale and methods for a participant-randomized effectiveness-implementation hybrid type II pilot trial designed to: 1) examine the effectiveness of Cognitive Processing Therapy group (CPT), an evidence-based psychotherapy for posttraumatic stress disorder (PTSD), for reducing post-release drug use and PTSD symptoms when adapted for and delivered in prisons; and 2) provide data on implementation outcomes associated with the use of implementation facilitation as a strategy for supporting uptake of CPT in prisons.

**Method:**

Participants in the effectiveness portion of the trial (*N* = 120) will be incarcerated men and women who are randomly assigned to one of two group therapies: CPT or a control condition (PTSD coping skills group; PCS). Participants will complete assessment measures three times: pre-treatment, post-treatment, and 3 months following release from incarceration. CPT groups will be led by prison counselors who are receiving implementation facilitation to support their efforts. PCS groups will be led by trained clinicians on the research team. Implementation outcomes will include acceptability, appropriateness, adoption, feasibility, fidelity, and sustainability. After enrollment ends, the research team will monitor CPT sustainment and recidivism outcomes of study participants for one year.

**Discussion:**

This study will lay the groundwork for a larger study of interventions for co-occurring PTSD and SUD in prisons and, critically, inform the development of strategies (such as implementation facilitation) for supporting their uptake in routine practice.

**Trial registration:**

NCT04007666, clinicaltrials.gov, 24 June 2019, 02 September 2021.

## Introduction

Collectively, US prisons do not provide effective behavioral health services to the 2.17 million people (1 in 110 US adults; Kaeble & Glaze, 2016) that they house. This is evident in that people who have been incarcerated are 13 times as likely to die in the two weeks after release as the general population, largely due to extreme rates of overdose (Binswanger et al., 2007; Merrall et al., 2010; Mital et al., 2020; Pizzicato et al., 2018; Spaulding et al., 2011). This suggests that many existing prison-based substance use disorder (SUD) treatment programs are inadequate. There is thus an urgent need to identify interventions to effectively reduce post-release drug use and to identify implementation strategies to support uptake by prison-based providers.

### Need for PTSD treatment in prisons

Evidence-based therapies to treat PTSD symptoms are under-explored interventions for reducing post-incarceration drug use. PTSD and SUDs frequently co-occur (Grant et al., 2015, 2016) and can synergistically exacerbate poor health outcomes (Back et al., 2000; Mccauley et al., 2012; Meshberg-Cohen et al., 2021; Simpson et al., 2019). PTSD development predicts the onset of drug use (Breslau et al., 2003) and PTSD symptom severity is linked to drug use severity (Back et al., 2000; Tripp et al., 2020). PTSD symptoms can also acutely motivate drug use (Hawn et al., 2020; Weiss et al., 2022) and lead to drug cravings (Farrelly et al., 2021; Vujanovic et al., 2019). In addition, both drug use and PTSD symptoms appear to decrease following integrated trauma-focused therapies (Roberts et al., 2015; Simpson et al., 2021).

The prevalence of drug use and trauma exposure among people in prison exceeds 80% (Bronson et al., 2017; Harner et al., 2015; Komarovskaya et al., 2011; Wolff et al., 2014). People who are incarcerated also have disproportionate prevalence of trauma sequelae including double the rates of PTSD and over ten times the rates of SUD as same-gender community peers (Baranyi et al., 2018; Fazel et al., 2017; Karlsson & Zielinski, 2020; Trestman et al., 2007). Indeed, PTSD has emerged as a risk factor for incarceration and predicts poorer post-release outcomes (e.g., relapse, recidivism; Anderson et al., 2016; Jäggi et al., 2016; Kubiak, 2004; Sadeh & McNiel, 2015).

### Strategies to support PTSD treatment uptake in prisons

Implementation facilitation (IF) emphasizes the development of supportive interpersonal relationships through which implementation strategies that are specifically matched to the needs of the intervention, context, and recipients can be delivered. Although IF has not been evaluated in prisons, it is evidence-based in supporting intervention uptake in complex systems (Baskerville et al., 2012; Harvey et al., 2002; Kirchner et al., 2014; Stetler et al., 2006) and is unique in its ability to be carefully tailored to the target setting(s). Thus, IF may be a promising strategy to support PTSD treatment uptake in prisons.

### Pilot trial overview

This article describes the study protocol of a pilot participant-randomized hybrid type II trial to assess effectiveness and implementation outcomes of group CPT—a gold-standard talk therapy for PTSD—that has been adapted for implementation in prisons (CPT-Criminal Justice Version, or CPT-CJ). We will also preliminarily explore the utility of IF to support prison counselors in delivering CPT-CJ with fidelity and will document discrete implementation strategies that are used as a part of IF.

## Methods

This study is funded by the National Institute on Drug Abuse. It was approved by the Institutional Review Board at the University of Arkansas for Medical Sciences and by the Office for Human Research Protections of the U.S Department of Health and Human Services. A four-member Data Safety Monitoring Board has been assembled to monitor the trial, which is registered at clinicaltrials.gov (NCT04007666).

### Study design and rationale

This study is a pilot effectiveness-implementation hybrid type II participant-randomized controlled trial which will compare IF-supported CPT-CJ (i.e., group-delivered CPT that has been adapted for implementation in prisons) to PTSD Coping Skills Group (PCS; a control intervention) in two Arkansas prisons. “Hybrid” effectiveness-implementation trials are used to study promising interventions and implementation strategies concurrently (Curran; et al., 2012). The Hybrid Type II design both emphasizes effectiveness and implementation outcomes; it is used when there is a strong base of indirect evidence suggesting—but not guaranteeing—applicability to a new setting/population for both the intervention and the implementation strategy. One primary aim of Hybrid Type II trials is to examine the effectiveness of a clinical intervention. A co-primary aim is to examine the feasibility and potential utility of one or more implementation strategies. Because both CPT and IF are effective in other settings, a hybrid type II design was deemed appropriate for this trial. Elements of the trial (including the comparison condition, CPT-CJ training format, and implementation strategies designed to overcome barriers) were selected in collaboration with study stakeholders during a series of four pre-trial Evidence-Based Quality Improvement sessions.

### Study setting

The proposed study will take place in two Arkansas prisons that are organizationally housed within Arkansas Division of Community Corrections. The facilities are single-sex, licensed as substance use treatment centers by the state of Arkansas, and house approximately 250–350 residents. The sites were selected with the assistance of the Division of Community Correction’s Resident Programming Director due to proximity to one another and opportunity to contribute additional programming. Most residents are non-Latinx and White per state-reported data. Most residents are incarcerated on felony drug and/or financial charges and serving sentences of 3 years or less. Residents are generally eligible for parole after serving one-third of their sentence, but ultimately release depends on a variety of factors including program participation and disciplinary write-ups that may occur during incarceration.

### Participant recruitment and assessment

Participants will include people who are incarcerated as well as prison stakeholders. Incarcerated persons will be randomly selected for participation from those who express interest and are eligible, except that our process will prioritize study entry for non-Latinx White participants given the limited racial and ethnic diversity at our study sites. Prison stakeholders will be purposively sampled based on their role in implementation of CPT and other programs.

#### Incarcerated persons

##### Recruitment and enrollment

Incarcerated participants will include men (*n* = 60) and women (*n* = 60) who have a pre-incarceration history of substance use and ongoing difficulties related to trauma. They will be eligible to participate in the study if they are/have: 1) at least 18 years old; 2) used drugs and/or alcohol in the year prior to becoming incarcerated; 3) a history of an SUD and/or addiction treatment; 4) a history of trauma; 4) clinically significant PTSD symptoms (score ≥ 3 on the *Primary Care PTSD Screen for DSM-5*; (Prins et al., 2016); 5) fluent in English; 6) able to consent; and 7) expected to be incarcerated long enough to complete all sessions of the intervention. Participants will have the opportunity to complete screening measures assessing their eligibility based on the aforementioned criteria throughout their incarceration. The screening measures will be offered upon intake to the facility by correctional staff; they may also be completed at any time during incarceration should an individual later request to complete it or should a counselor refer particular individuals to the study.

##### Randomization

**Fig. 1:**
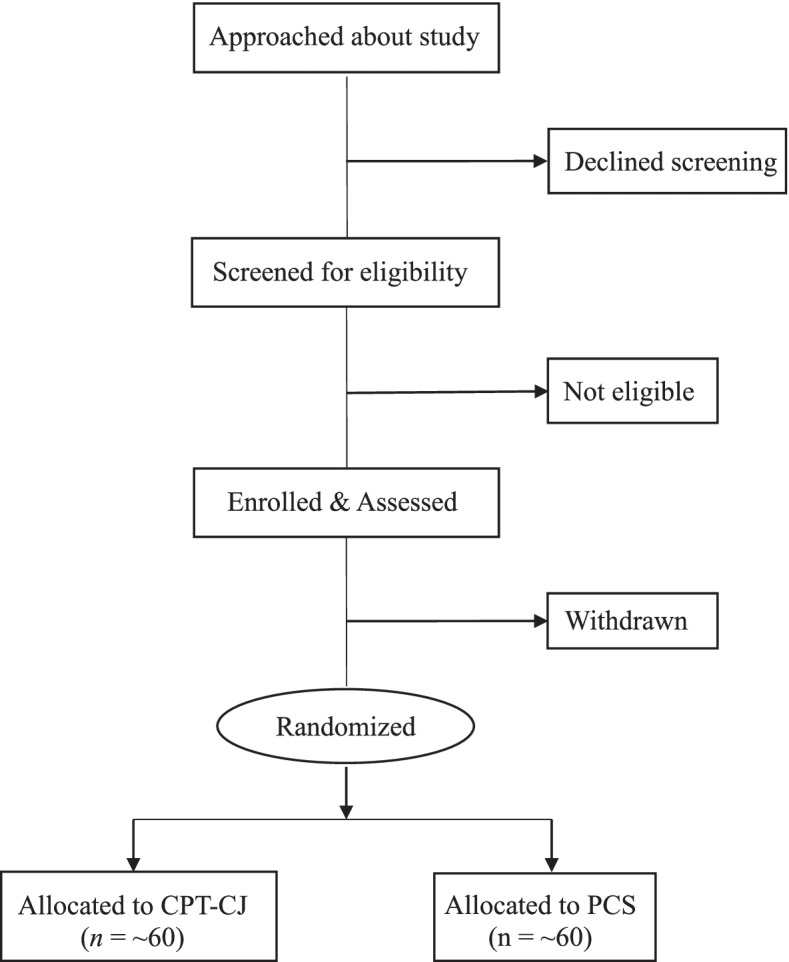
Study CONSORT Diagram

Randomization will occur separately by site, which are single-sex, after pre-treatment assessment. Pairs will be formed based on age (under 30 vs. age 30 or older), race-ethnicity (non-Latinx White vs. any other race/ethnicity), and drug use severity (mild to moderate vs. severe symptoms). One member of each pair will be assigned to each treatment arm. Individuals who cannot be paired (i.e., those without a close match on age, race-ethnicity, and drug use severity) in a given round of recruitment will be randomly assigned to one of the conditions if the treatment arms are not already filled. Only the project PI and project Biostatistician will be aware of group assignments; the rest of the project team will remain blinded to treatment condition. Figure 1 depicts the study flow.

##### Administrative data

Descriptive data on treatment attendance will be tracked from administrative records (e.g., sign-in sheets), which will allow us to assess the intervention dose and frequency.

##### Assessment schedule

Incarcerated persons will be assessed at three time points: 1) pre-treatment; 2) post-treatment; and 3) three months post-release. See Table 1 for assessment measures. All assessments will be conducted by trained research staff who are blinded to participant treatment condition. Pre-treatment assessments of eligibility and baseline symptoms will be completed within approximately four weeks of treatment start. Post-treatment assessments will be completed within approximately three weeks after treatment ends. Post-release assessments will occur approximately three months after release to determine whether effectiveness outcomes are maintained and if participants in the two arms differ on post-release drug use. Post-release assessments will generally be conducted in the community via phone or televideo; however, they may be conducted in carceral or other residential settings (e.g., substance use treatment centers) if that is where the participant resides. Recidivism data are administratively recorded by the correctional facility and will be obtained for analyses at the end of the trial.

**Table 1:** Self-Report Measure Administration Schedule

Measure	Pre-Treatment	During Treatment	Post-Treatment	Post-Release
Primary Care Screen for PTSD	▪		▪	▪
Life Events Checklist ^a^	▪		▪	▪
PTSD Checklist for DSM-5	▪	▪	▪	▪
Patient Health Questionnaire–9-item	▪	▪	▪	▪
Posttraumatic Cognitions Inventory–9-item	▪		▪	▪
Clinician-Administered PTSD Scale for DSM-5	▪		▪	▪
Multidimensional Scale of Perceived Social Support	▪		▪	▪
Outcome Rating Scale	▪		▪	▪
Timeline Follow Back Interview Calendar	▪		▪	▪
Alcohol and Drug Use Questionnaire	▪			▪
DSM-5 Checklist of Alcohol Use Disorder Criteria	▪			▪
DSM-5 Checklist of Drug Use Disorder Criteria	▪			▪
Acceptability of Intervention Measure	▪			
Intervention Appropriateness Measure	▪			
University of Rhode Island Change Assessment	▪			
Selected Personality Assessment Inventory Subscales	▪			
Distress Tolerance Scale	▪		▪	
Personal Feelings Questionnaire	▪		▪	
Brief Self-Control Scale	▪		▪	
Difficulties in Emotion Regulation Scale–Short Form	▪		▪	
Brief Inventory of Psychosocial Functioning	▪		▪	
Dimensions of Anger Reactions–5-item	▪		▪	
Client Satisfaction Questionnaire			▪	
Post-Treatment Feedback Form ^b, c^			▪	
Helping Alliance Questionnaire–Revised			▪	
Recovery Capital Scale				▪
Posttrauma Risky Behaviors Questionnaire				▪
Healthcare Utilization Survey ^b^				▪
Qualitative Questions ^b^				▪

^a^ Modified from original format to obtain focused information on trauma frequency, age at exposure, and exposure relative to incarceration

^b^ Created for use in this study

^c^ The Post-Treatment Feedback Form queries for incarcerated persons’ reactions to the therapy that they received including but not limited to their perceptions of: the impact of the therapy; the most and least helpful aspects of the therapy; whether the therapy had any negative impact on them; and whether they would recommend the therapy to other incarcerated people

##### Retention

Participants will be asked to provide information that will help the study team to stay in contact with them during the pre- and post-treatment assessment and again shortly after release from incarceration. At each of these contacts, they will be asked to confirm or update their expected direct contact information (i.e., phone number, address, email). They will also be asked for potential alternate contact methods to use if attempts to reach them through their direct contact information are unsuccessful. Alternate contact methods may include social contacts, organizations, social media profiles, and/or parole offices.

Study staff will seek to maintain regular contact with participants using these contact methods to facilitate completion of the post-release assessment. Methods used will include: calling, texting, email, mail, and/or direct messaging on social media with participants and/or with individuals and organizations that they have identified as likely to be able to help with locating them if needed. Publicly-available information (e.g., court records, jail roasters) may also be used to locate and maintain contact with participants.

##### Primary and secondary effectiveness outcomes

The primary effectiveness outcomes for this pilot trial are post-release drug use and PTSD symptoms. Post-incarceration drug use will be assessed via Timeline Follow-Back Interview and examined as both binary responses (abstinence vs. any use) and frequency counts (number of days of use). PTSD symptoms will be assessed using the 20-item PTSD Checklist (PCL-5; Weathers et al., 2013). Lower scores indicate lower levels of PTSD symptoms and therefore a better treatment outcome. Depression symptoms, recidivism, and more general post-release functioning (e.g., social, occupational, financial) will be assessed as secondary outcomes. Depression symptoms will be assessed using the Patient Health Questionnaire (PHQ-9). Recidivism will be extracted from administrative incarceration records and examined as both a binary variable (presence or absence of any drug-related recidivism) and frequency counts (number of new drug charges or convictions). Drug-related recidivism will be defined as 1) new arrests for drug charges and 2) new convictions for drug charges; each will be examined separately. All other measures are being administered to allow for the assessment of possible mediators and moderators of treatment effects or are exploratory.

#### Prison stakeholders

Prison stakeholders (i.e., direct service providers and systems-level administrators involved in implementation; anticipated *n* = 7 per site) will complete brief surveys and qualitative interviews two times during the trial. Interviews will primarily examine implementation determinants and will be guided by the Consolidated Framework for Implementation Research (CFIR; Damschroder & Hagedorn, 2011). For example, interviewees will respond to questions about factors such as CPT-CJ compatibility with the setting, resources, and population; personal perceptions of CPT-CJ; leadership engagement in implementation; and the role of external policies and initiatives. Counselors who offered CPT-CJ groups as a part of the trial will also be asked to provide feedback on the strategies that were used to support CPT-CJ implementation, with a particular focus on strategies that aimed to train them in the intervention. Interviews will typically be conducted once after enrollment ends and 12-months later, which will be the end of the sustainment monitoring period. However, should a stakeholder leave their role in the project (e.g., resign, transfer), they will be invited to participate in the interview and surveys at that time. This data will provide a nuanced picture of providers’ feelings about CPT-CJ and the IF strategy, and will inform alterations to the treatment and implementation strategies.

### Intervention conditions

#### Cognitive Processing Therapy – Criminal Justice version (CPT-CJ)

##### CPT-CJ overview

CPT is a gold-standard evidence-based psychotherapy for PTSD that combines education about trauma with strategies to challenge the trauma-related cognitions that are theorized to maintain PTSD symptoms (Resick et al., 2017). Over the last 40 years, CPT has been shown to reduce PTSD and other outcomes of trauma, such as depression, anxiety, insomnia, and dysregulation (Chard, 2005; Clarke et al., 2008; Galovski et al., 2005, 2009; Monson et al., 2006; Resick et al., 2002). CPT has also proven effective in settings characterized by ongoing violence, limited privacy, and housing instability (e.g., conflict zones, refugee camps), which share similarities with prisons (Bass et al., 2013; Bolton et al., 2014; Greene et al., 2019; Kaysen et al., 2013, 2020). CPT has also been well-tolerated by individuals with SUD in prior trials (Held et al., 2021; Kaysen et al., 2014; Pearson et al., 2019). To our knowledge, there is only one study that examined the use of CPT with incarcerated populations. In a sample of incarcerated adolescent boys, Ahrens and Rexford (2002) found that those in the CPT group had less PTSD and depression at post-treatment when compared to the wait list group.

CPT was modified for implementation in prisons for this trial based on the results of formative evaluation conducted with the two study sites. All modifications were fidelity-congruent. A full description of these modifications are beyond the scope of this paper, but generally modifications consisted of 1) design changes to handouts and worksheets to add visual cues to pair with verbal concepts, 2) altering examples on illustrative handouts and worksheets to better fit the acute and historical experiences of the population, 3) adapting the therapist manual with language consistent with running the group in a prison and procedures specific to handling common occurrences that are specific to prisons (e.g., turning their radio down at the start of group), and 4) instructions to have a more extended discussion of confidentiality, group norms, and how typical facility rules do or do not apply as a part of the first group session. The modified protocol (CPT-CJ) will be the intervention condition evaluated in this trial. CPT-CJ will be delivered in a group format because group treatment is a familiar and feasible treatment modality in most prison settings. Because CPT is robust to number (Fleming et al., 2020; Galovski et al., 2012, 2021; though also see Gutner et al., 2016), duration, and frequency of sessions, structure will be allowed to flex, with the goal being to remain within the range evaluated in prior research (i.e., include 8–12 sessions, occur 1–2 times per week, last 60–90 min) based on facility considerations.^1^

##### Providers and provider training and consultation

Counselors selected by each site will attend a virtual training in CPT-CJ. This virtual training will be approximately 16-h and be led by certified CPT providers. Following the training, counselors will attend weekly consultation with a certified CPT provider for at least two groups. Consultation may be extended for a longer duration if desired by the group leaders or if determined to be necessary by the PI to promote fidelity. The counselors will also complete at least 1 run-in group prior to the start of the research study during which they will receive more intensive supervision, including the study PI live observing CPT-CJ group sessions. The providers at the start of the trial will be bachelor’s- and/or master’s-level counselors who have degrees in psychology, social work, or a related field and who are already employed by the correctional facility to provide individual and group counseling.

##### Fidelity

To promote and monitor fidelity, the PI will attend CPT-CJ sessions at each site via televideo and sit in on all sessions at the start of the trial. Additional methods to support fidelity will be incorporated if needed as part of IF (e.g., strategies such as audit and feedback). Fidelity will be quantified as a ratio of the number of intervention components planned versus the number delivered each session. Fidelity data will be obtained from structured session checklists that will be completed while observing sessions.^2^

#### Coping-focused skills group as an enhanced standard of care control condition

The goals of the comparison condition selection were: 1) to select a group similar to one that prisons may choose to run if they had independently decided to initiate trauma-focused treatment outside of a research trial; and 2) to approximately match for attention and dose without adding additional costs to the system. During formative evaluation, it was revealed that both project sites had previously provided coping-focused programming and coping-skill approaches to trauma treatment. Coping-focused interventions are common alternatives to trauma-focused therapies such as CPT (which deal more directly with the index trauma).

To provide an enhanced standard of care, the PI reviewed treatment materials already used in prisons and similar communal-living settings and the research literature on coping-focused skills groups that had been evaluated in prisons in past research trials. The PI used this review to outline three potential comparison treatment group options and present them to a stakeholder panel consisting of treatment staff at the participating sites. The stakeholder group selected for the comparison arm intervention to be a moderately structured group focused on teaching a coping-focused curriculum of skills. The group will be based on a self-help workbook (Tull et al., 2017) and referred to as “PTSD Coping Skills Group” (PCS) in the trial. The intervention will focus on helping people to cope with symptoms related to PTSD instead of directly addressing the traumatic event at length. Providers for this intervention will be clinical research team members under the supervision of the PI and/or the PI herself.

### Implementation Facilitation (IF) strategy

IF will support the uptake of CPT-CJ at both sites. As part of IF, the PI will serve as an external facilitator and work closely with an internal champion at each site. Because facilitation is a dynamic process and individual implementation strategies may be added as needed during the trial to promote implementation, the PI will: 1) maintain tracking logs of all IF activities and 2) conduct qualitative debriefings to document implementation strategies applied. We expect the trial IF strategies to include at least the following: pre-implementation strategies such as engaging leadership; identifying key stakeholders; and academic detailing of clinicians; early- to mid-phase strategies such as monitoring and promoting implementation guide execution; identifying and addressing barriers; keeping leadership informed/engaged; monitoring fidelity and intervening if needed; and adapting the implementation guide; and late-phase implementation strategies such as audit and feedback and role modeling.

### Implementation outcomes

Selection of the implementation outcomes was guided by Proctor et al.’s (2011) conceptual framework for implementation research. Our implementation outcomes for the pilot trial will thus be acceptability, appropriateness, adoption, fidelity, feasibility, and sustainability. *Adoption* will be calculated as the ratio of the number of incarcerated persons who complete CPT-CJ to the number who were randomized to it. *Fidelity* will be calculated as the ratio of the number of CPT-CJ components delivered to the number of CPT-CJ components planned. Provider competence in delivering each element will also be calculated as an average across components on a scale ranging from 1 (*Poor*) to 5 (*Excellent*). *Sustainability* will be measured in the final year of the trial through monitoring of CPT-CJ enrollment and fidelity. Sites will be considered to have sustained CPT-CJ provision if they maintain or increase the number of incarcerated persons who complete CPT-CJ over the 12-month monitoring period after enrollment has ended. *Acceptability* and *feasibility* will be measured based on the perspectives of systems-level administrators, providers, and participants through self-report surveys (Weiner et al., 2017) and within qualitative interviews.

### Sample size justification and data analysis plan

#### Power analysis

Statistical power for the pilot trial was assessed using the two primary effectiveness outcomes measured at post-release assessment: binary drug use (abstinence vs. any use, per the Timeline Follow-Back Interview) and PTSD symptoms (sum score on the PCL-5). G*Power 3.1.9.2 and Optimal Design Software were used for power calculations. For each outcome we assume a two-sided α = 0.05 for a two independent groups (i.e., CPT-CJ vs. PCS) comparison. For drug use, the anticipated sample of *n* = 86 (adjusted due to projected attrition) will provide 80% power to detect a difference of 27.2 percentage points on drug abstinence between groups assuming a rate of 60% post-prison drug use in the PCS group (Kinlock et al., 2008). For PTSD, this same sample will provide 80% power to detect a difference of 0.57 standard deviations. Based on published work which measured incarcerated adults’ PTSD symptoms with the PCL-C (the previous version of the PCL-5), we anticipate a *SD* of 11.0 points on the PCL-5 (Decou et al., 2015; Wolff et al., 2014). Thus, the pilot trial is powered to detect a change of approximately 6.3 points. For context, a 5-point change has been described as a reliable change and a change between 10 and 20 points is indicative of a clinically significant change (U.S. Department of Veterans Affairs, 2014).

#### Analysis of effectiveness outcomes

Intention-to-treat analyses on primary (drug use and PTSD) and secondary (depression and drug-related recidivism) outcomes will be used to evaluate CPT-CJ’s effectiveness in comparison to the PCS group at post-treatment and at post-release follow-up. Examination of response variable distributions will inform choice of a link function for generalized linear models, which accommodate response variables with different distributions and allow for inclusion of relevant covariates. Drug-related outcomes will be examined as both binary responses (abstinence vs. any use; presence or absence of drug-related recidivism) and frequency counts (number of days use; number of new drug-related crimes). PTSD and depression symptom scores will be examined as continuous responses.

##### Sex differences

Equal numbers of men and women will participate in this trial. Thus, sex differences in the data collected will be examined at each time point for the purpose of hypothesis generation and informing future trials. After treatment, it will be possible to preliminarily examine sex-by-treatment interactions, providing an indicator of whether treatments are differentially effective by sex.

##### Nested data

The intervention is delivered in groups, therefore responses within groups may be correlated (Raudenbush & Bryk, 2002; Selig et al., 2017). Due to potential correlation, we will examine each of the response variables for the possibility of nesting using the intraclass correlation. For response variables showing a notable degree of nesting, we will employ mixed effects/multilevel models that can accommodate nested data.

#### Analysis of implementation outcomes

The CPT-CJ implementation outcomes in will be summarized descriptively. Qualitative results will be summarized via rapid coding based on CFIR domains (i.e., inner setting, outer setting, intervention characteristics, characteristics of individuals, process). We will then inductively code text in each domain with descriptive sub-codes that represent the barriers and facilitators to CPT-CJ implementation noted in each domain.

## Results

Not applicable.

## Discussion

This study will increase knowledge on: 1) strategies for implementing trauma therapies in prisons; and 2) the effectiveness of a particular therapy, CPT-CJ, when delivered in prisons. With regard to the latter, this study will be the first to examine whether CPT-CJ reduces drug use and recidivism in adults after release from incarceration. Increasing access to proven trauma therapies in prisons may reduce drug use, crime, costs, and community burden associated with incarceration by improving prisoners’ mental health prior to release.

Importantly, this trial was developed in close partnership with stakeholders at the study sites. Using an evidence-based quality improvement process (described elsewhere) we consulted with stakeholders about many of the design decisions including when originally selecting CPT as the intervention, modifying CPT for prisons (as described above), developing our CPT-CJ implementation plan, selecting trial outcomes, and selecting the comparison group. We also rigorously evaluated anticipated barriers and facilitators to CPT-CJ implementation prior to the start of the trial and have adjusted our plans based on this feedback. Together, our hope is that this approach will result in the pilot trial having relatively high external validity in prisons similar to our pilot sites (e.g., more therapeutically oriented prisons). On the other hand, research trials bring resources to sites that would be otherwise unavailable and thus the conduct of research itself may facilitate implementation; for example, the trial is able to purchase materials for the sites and pay for training and supervision in the intervention. This differs from real-world conditions wherein agencies that desire to bring in new interventions must pay these costs. In this trial, we will be able to monitor for sustainability once research support is withdrawn but we will not be able to assess barriers/facilitators that would have arisen if our partnering sites had needed to implement CPT-CJ without expert support. Additional limitations of the trial are the small number of sites and limited racial and ethnic diversity of the sample of incarcerated participants. While we will attempt to address the latter by prioritizing people who are non-White and/or Latinx for inclusion, it is likely that our final sample will still be heavily non-Latinx and White. Notably, this is not the case for study stakeholders, including study therapists, who have significantly more racial diversity. A larger trial with more prisons that vary more in terms of physical structure, norms, and facility populations will also be needed to more robustly assess external validity and to identify challenges that may arise during scale up.

## Conclusion

If effective, wide-scale implementation of evidence-based therapies for PTSD, such as CPT-CJ, in prisons may improve outcomes of incarcerated Americans who have co-occurring drug use and PTSD symptoms. If results of this pilot trial are promising, we anticipate that a follow-up trial with a focus on replicating the effectiveness results and refining the implementation strategy would be warranted.

## Data Availability

Not applicable.

## Abbreviations

CPT: Cognitive Processing Therapy
CPT-CJ: Cognitive Processing Therapy—Criminal Justice Version
IF: Implementation facilitation
PCS: PTSD Coping Skills Group
PTSD: Posttraumatic stress disorder
SUD: Substance use disorder

## Footnotes

1 Including any limitations on movement imposed by the ongoing COVID-19 pandemic.

2 Checklists adapted from “Cognitive Processing Therapy (CPT) Therapist Adherence and Competence Protocol” by Macdonald, Wiltsey-Stirman, Wachen, & Resick (2014) based on original work titled “Cognitive Processing Therapy – Cognitive Only” by Birks, Rodgers, and Morland.
